# Correspondence: Reply to ‘Compound 17b and formyl peptide receptor biased agonism in relation to cardioprotective effects in ischaemia-reperfusion injury’

**DOI:** 10.1038/s41467-017-02656-0

**Published:** 2018-02-07

**Authors:** Cheng Xue Qin, Lauren T. May, Patrick M. Sexton, Aaron J. DeBono, Jonathan B. Baell, Arthur Christopoulos, Rebecca H. Ritchie

**Affiliations:** 1 Baker Heart and Diabetes Institute, 75 Commercial Road, Melbourne, VIC 3004 Australia; 2 Department of Pharmacology and Therapeutics, University of Melbourne, Melbourne, VIC 3010 Australia; 3 Drug Discovery Biology and Department of Pharmacology, Monash Institute of Pharmaceutical Sciences, Monash University, 381 Royal Parade, Parkville, VIC 3052 Australia; 4 Medicinal Chemistry, Monash Institute of Pharmaceutical Sciences, Monash University, 381 Royal Parade, Parkville, VIC 3052 Australia; 5 School of Pharmaceutical Sciences, Nanjing Tech University, No. 30 South Puzhu Road, Nanjing, 211816 People’s Republic of China; 6 Department of Diabetes (Central Clinical School), Monash University, Melbourne, VIC 3004 Australia

## Introduction

Biased agonism describes the ability of an agonist to stabilise a unique subset of G protein-coupled receptor (GPCR) conformations compared to a reference agonist (for example, the endogenous agonist)^1^. This emerging paradigm is currently revolutionising drug discovery across a broad spectrum of disease pathologies, including those affecting the respiratory and central nervous systems, metabolism, inflammation and the heart^2–5^, to name but a few. This phenomenon thus provides the opportunity to potentially promote desired on-target signal transduction whilst eschewing possible detrimental effects^1^.

We recently reported that biased signalling is evident at formyl peptide receptors (FPRs), based on contrasting signalling fingerprints of two small-molecule FPR agonists in engineered Chinese Hamster Ovary (CHO) cells stably expressing human FPR1 and FPR2^6^, a family of GPCRs that regulate host defence, inflammation and its resolution^7,8^. Cmpd17b (*N*-(4-bromophenyl)-2-[5-(3-methoxybenzyl)-3-methyl-6-oxo-6H-pyridazin-1-yl]-propionamide) is a racemic compound first described by Cilibrizzi et al.^9^ Utilising a conventional FPR agonist Amgen compound-43 (Cmpd43)^10^ as the reference agonist, and Akt1/2/3(Thr308) phosphorylation as the reference pathway, we demonstrated that Cmpd17b exhibited approximately a 30-fold bias away from Ca^2+^ mobilisation relative to Cmpd43 at both FPR1 and FPR2. This was associated with differential pathophysiological outcomes, whereby Cmpd17b (but not Cmp43) mediated a spectrum of cardioprotective benefit both in vitro and in vivo^6^.

The recent correspondence from Cilibrizzi^11^ regarding our work provides a brief commentary regarding potential differences between our work and others with respect to FPR binding affinity, and whether we had considered the relevance of chirality with respect to FPR bias. We are pleased to see this interest in our work and are happy to respond to these comments. First, it is absolutely vital to make an important distinction between binding affinity, which is consistently referred to by Cilibrizzi throughout their correspondence^11^, and agonist potency, which is a measure of agonist activity in a cellular assay (e.g., calcium mobilisation) and commonly defined by the concentration required to stimulate 50% of the agonist response (EC_50_). Binding affinity is a true thermodynamic measurement and should be independent of cell background, as it is a *constant* for a given ligand–receptor complex. In contrast, agonist potency is a completely empirical parameter that is a composite value determined by not only binding affinity, but also agonist ‘intrinsic’ efficacy (the ability of a ligand to promote an active receptor conformation linked to a given pathway), receptor expression (the number of available receptors) and efficiency of stimulus-response coupling (the strength with which of each activated receptor couples to a particular signalling pathway)^12^. Because the latter properties are system-dependent (i.e. cell type) variables, it is absolutely of no surprise that agonist potency can vary from cell to cell^1^ and, in this regard, no particular recombinant cell type is superior to another. We agree with Cilibrizzi that reproducibility between different groups requires similar cell backgrounds and experimental settings, but this is because most GPCR screening assays in the field are cell-based functional assays^1^, yielding EC_50_ (not affinity) values that can vary for the reasons outlined above. Indeed, at no stage did our study determine agonist binding affinities; only agonist potencies. Similarly, all the values highlighted in Cilibrizzi Table 1^11^ are not agonist binding affinities but, rather, agonist potencies (EC_50_ values, as clearly stated in the table heading by Cilibrizzi but surprisingly referred to as ‘affinity’ values in the footnotes^11^). Thus, to address this issue and cancel the impact of system variables on drug-receptor properties in a manner that can transcend the cell type used for investigation, it is necessary to use a reference ligand (in the same cell background that is subject to the same system variables) against which all novel ligands are compared. For the study of biased agonism, this is even more important as it requires assessment across a range of different signalling pathways. The methods we used throughout our study are null methods, because they standardised all measures of agonist activity using a common cell background, a reference agonist and a reference pathway^6^. application of the Black-Leff operational model^13,14^, which incorporates both agonist binding affinity and signalling efficacy, to such data automatically eliminates the influence of system-dependent parameters for a given cell background, allowing the remaining differences to reflect ‘true’ biased agonism relative to the reference agonist.

**Table 1:** The activity of formyl peptide receptor agonists, potency (pEC_50_) and the response observed at 10 μM for each agonist (% positive control), for Ca^2+^_i_ mobilisation and ERK1/2 phosphorylation in human FPR-expressing FlpIn-CHO cells

	hFPR1				hFPR2			
	Ca^2+^_i_		pERK1/2		Ca^2+^_i_		pERK1/2	
	pEC_50_	10 μM response (%)	pEC_50_	10 μM response (%)	pEC_50_	10 μM response (%)	pEC_50_	10 μM response (%)
Cmpd43^a^	8.1 ± 0.1	42 ± 5^b^	7.1 ± 0.1	80 ± 7	7.8 ± 0.1	51 ± 3^b^	7.1 ± 0.1	95 ± 3
(±)-Cmpd17b	5.1 ± 0.1	22 ± 4	6.0 ± 0.1	70 ± 6	<5	6 ± 5	5.8 ± 0.1	82 ± 2
R-(−)-Cmpd17b	5.4 ± 0.1	38 ± 1	6.1 ± 0.1	82 ± 6	<5	8 ± 4	5.9 ± 0.1	85 ± 2
S-(+)-Cmpd17b	<5	16 ± 2	5.9 ± 0.1	78 ± 5	<5	4 ± 7	<5	8 ± 4^b^

Data represents the mean ± S.E.M. of 3–7 separate experiments, each conducted in duplicate or triplicate

^a^ Values determined from previously published data^6^

^b^ Significantly different, *P* < 0.05 compared to the corresponding 10 μM response (% positive control) value for (±)-Cmpd17b, one-way ANOVA with Dunnett’s post hoc analysis

**Fig. 1:**
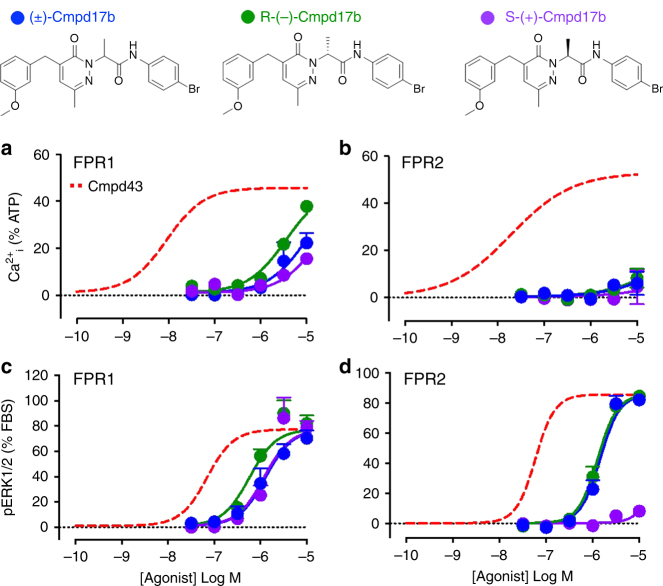
Signalling fingerprint of R,S-(±)Cmpd17b and its enantiomers in CHO cells stably expressing hFPRs. Influence of R,S-(±)Cmpd17b (blue), R-(−)Cmpd17b (green) and S-(+)Cmpd17b (purple) on intracellular signalling intermediates downstream of GPCRs. Concentration–response curves to Ca^2+^_i_ mobilisation at **a** hFPR1 and **b** hFPR2. Concentration–response curves to ERK1/2 phosphorylation at **c** hFPR1 and **d** hFPR2. Responses to all three compounds were determined concomitantly, with *n* = 3–7 separate experiments, each performed in duplicate or triplicate. Results are expressed as mean ± S.E.M. The red dashed line illustrates non-linear regression for the reference FPR agonist Cmpd43 from our original publication^6^. The chemical structure of Cmpd17b (*N*-(4-bromophenyl)-2-[5-(3-methoxybenzyl)-3-methyl-6-oxo-6H-pyridazin-1-yl]-propionamide), illustrating the position of the chiral centre, is shown on the upper panel

Second, Cilibrizzi raises a very important consideration regarding ligand chirality. We now demonstrate that each of R,S-(±)Cmpd17b, S-(+)Cmpd17b and R-(−)Cmpd17b stimulate concentration-dependent increases in intracellular Ca^2+^ (Ca^2+^_i_) mobilisation in hFPR1-CHO cells, but to a markedly more modest extent than previously demonstrated for the conventional FPR agonist Cmpd43 (Fig. 1a). The increases in Ca^2+^_i_ mobilisation stimulated by each of R,S-(±)Cmpd17b, S-(+)Cmpd17b and R-(−)Cmpd17b are ~1000-fold less potent than that observed in response to Cmpd43 at hFPR1 (Table 1). The stimulation of Ca^2+^_i_ mobilisation in hFPR2-CHO cells was similarly modest for each of the three small molecules, at least 600-fold less potent than Cmpd43 (Fig. 1b; Table 1). Each of R,S-(±)Cmpd17b, S-(+)Cmpd17b and R-(−)Cmpd17b also stimulate concentration-dependent increases in ERK1/2 phosphorylation in hFPR1-CHO cells (Fig. 1c). At the hFPR1, R,S-(±)Cmpd17b and each of its enantiomers, S-(+)Cmpd17b and R-(−)Cmpd17b, stimulated ERK1/2 phosphorylation with a similar micromolar potency, ~10-fold less potent than Cmpd43 (Table 1). In contrast, the stimulation of ERK1/2 phosphorylation by R-(−)Cmpd17b was virtually superimposable on that observed in response to R,S-(±)Cmpd17b, with negligible activity of S-(+)Cmpd17b with respect to hFPR2-mediated ERK1/2 phosphorylation.

These new findings demonstrate that, in human FPR-expressing FlpIn-CHO cells, the activity of the two enantiomers, R-(−)Cmpd17b and S-(+)Cmpd17b, particularly with respect to ERK1/2 phosphorylation, are comparable to the responses obtained by Cilibrizzi^11,15^; supporting the different behaviour of the S-(+) enantiomer at FPR1 and FPR2. We agree that future studies should evaluate the signalling profile of the two enantiomers with respect to β-arrestin recruitment. The contrasting ability of the two enantiomers with respect to FPR activation is not unexpected given the spatial requirements of GPCR-agonist interactions. Indeed, Corbisier et al. have recently reported biased agonism for enantiomers at chemokine CCR2 and CCR5 receptors^16^. Our new observations suggest that R-(−)Cmpd17b, similar to racemic R,S-(±)Cmpd17b, is a biased agonist at hFPR1, eliciting activation of ERK1/2 phosphorylation in stably-transfected CHO cells (a pathway that is key to cardiomyocyte survival) yet biased away from Ca^2+^_i_ mobilisation (a pathway implicated in cardiomyocyte loss post ischaemic injury, as well as in inflammatory cell migration). In contrast, S-(+)Cmpd17b elicits negligible responses at both pathways downstream of FPR2. It is thus likely that R-(−)Cmpd17b may be responsible for a significant component of the R,S-(±)Cmpd17b cardioprotection observed in the intact heart over the longer term. Future examination of the effectiveness of R-(−)Cmpd17b compared to S-(+)Cmpd17b in the context of pre-clinical settings of myocardial infarction is thus warranted.

In conclusion, the methods used to quantify bias across a spectrum of FPR signalling pathways (in the case of our studies utilising R,S-(±)-Cmpd17b in engineered CHO cells, in which five signals were included in the bias quantification), incorporating a reference agonist and a reference pathway, remove the influence that might otherwise be posed by differences for a given cell background and, indeed, even ‘true’ differences in binding affinity (if such differences exist)^1^. Hence, we are confident that our conclusions from our original study^6^, that: (i) R,S-(±)-Cmpd17b is a biased agonist at FPR1 and FPR2, relative to the reference agonist Cmpd43; (ii) R,S-(±)-Cmpd17b promotes markedly less Ca^2+^_i_ mobilisation downstream of FPR1 and FPR2 relative to ERK1/2 signal transduction; and (iii) the biased FPR agonist R,S-(±)-Cmpd17b elicits superior outcomes following myocardial injury in mice in vivo are indeed justified. We contend that the resultant improved understanding of the mechanisms of small-molecule FPR agonists may facilitate targeted development of FPR-biased strategies for improving outcome after myocardial infarction.

## Methods

Cmpd17b (500 mg) was synthesised as per the original paper in which the biased profile for this racemic compound was reported^6^, using the method published by Cilibrizzi et al.^9^ Guided by the methods described by Cilibrizzi et al.^15^ chiral separation of the (+) and (−) enantiomers of Cmpd17b was performed by SYNthesis med chem (Parkville, VIC, Australia) via chiral HPLC to yield pure enantiomers. Concentration–response curves to both Ca^2+^i mobilisation and ERK1/2 phosphorylation were generated in stably-transfected recombinant hFPR-CHO cells in response to each of (±)Cmpd17b, (+)Cmpd17b and (−)Cmpd17b. Data were normalised to responses elicited by the relevant positive control, 100 µM ATP and 10% FBS, respectively, as described in the original manuscript^6^. All data were analysed using GraphPad Prism 7 (GraphPad Software). Results are expressed as mean ± S.E.M. of 3–7 experiments, conducted in duplicate or triplicate. Statistical analyses utilised one-way ANOVA followed by Dunnett’s post hoc test. Values of *P* < 0.05 were considered statistically significant.

### Data availability

The data that support the findings of this study are available from the corresponding author R.H.R. on request.
